# An Ultra-Processed Food Dietary Pattern Is Associated with Lower Diet Quality in Portuguese Adults and the Elderly: The UPPER Project

**DOI:** 10.3390/nu13114119

**Published:** 2021-11-17

**Authors:** Milena Miranda de Moraes, Bruno Oliveira, Cláudia Afonso, Cristina Santos, Duarte Torres, Carla Lopes, Renata Costa de Miranda, Fernanda Rauber, Luiza Antoniazzi, Renata Bertazzi Levy, Sara Rodrigues

**Affiliations:** 1 Faculty of Nutrition and Food Sciences, University of Porto, 4150-180 Porto, Portugal; bmpmo@fcna.up.pt (B.O.); claudiaafonso@fcna.up.pt (C.A.); cristinasantos@fcna.up.pt (C.S.); dupamato@fcna.up.pt (D.T.); saraspr@fcna.up.pt (S.R.); 2 Associated Laboratory ITR, Laboratory for Integrative and Translational Research in Population Health—Institute of Public Health, University of Porto, 4050-600 Porto, Portugal; carlal@med.up.pt; 3 Artificial Intelligence and Decision Support (LIAAD), Institute for Systems and Computer Engineering, Technology and Science (INESC TEC), 4200-465 Porto, Portugal; 4 Center for Health Technology and Services Research (CINTESIS), University of Porto/Associate Laboratory RISE—Health Research Network, 4200-450 Porto, Portugal; 5 Faculty of Medicine, University of Porto, 4200-319 Porto, Portugal; 6 Department of Nutrition, Institute of Health Sciences, Federal University of Triângulo Mineiro, Uberaba 38025-440, Brazil; renata.miranda@uftm.edu.br; 7 Center for Epidemiological Research in Nutrition and Health, University of São Paulo, São Paulo 01246-90, Brazil; rauber@usp.br (F.R.); luiza.antoniazzi@hotmail.com (L.A.); rlevy@usp.br (R.B.L.); 8 Department of Preventive Medicine, Faculty of Medicine, University of São Paulo, São Paulo 01246-90, Brazil; 9 Department of Nutrition, School of Public Health, University of São Paulo, São Paulo 01246-90, Brazil

**Keywords:** dietary patterns, latent class analysis, ultra-processed foods, diet quality, feeding behavior

## Abstract

This study aimed to identify dietary patterns (DPs) and their associations with sociodemographic factors and diet quality in Portuguese adults and the elderly. Cross-sectional data were obtained from the National Food, Nutrition and Physical Activity Survey (2015–2016), with two non-consecutive dietary 24 h recalls. Food items were classified according to the NOVA system and its proportion (in grams) in the total daily diet was considered to identify DPs by latent class analysis, using age and sex as concomitant variables. Multinomial logistic and linear regressions were performed to test associations of DPs with sociodemographic characteristics and diet quality, respectively. Three DPs were identified: “Traditional” (higher vegetables, fish, olive oil, breads, beer and wine intake), “Unhealthy” (higher pasta, sugar-sweetened beverages, confectionery and sausages intake) and “Diet concerns” (lower intake of cereals, red meat, sugar-sweetened and alcoholic beverages). “Unhealthy” was associated with being younger and lower intake of dietary fiber and vitamins and the highest free sugars and ultra-processed foods (UPF). “Diet concerns” was associated with being female and a more favorable nutrient profile, but both DPs presented a higher contribution of UPF than the “Traditional” DP. These findings should be considered for the design of food-based interventions and public policies for these age groups in Portugal.

## 1. Introduction

Non-communicable disease prevalence has been increasing worldwide, making diet-related diseases one of the leading risks for mortality and disabilities [1], and were the cause for 88% of the deaths in Portugal in 2017 [2]. A high body mass index (BMI) is an important risk factor for non-communicable diseases [3], and in recent decades the prevalence of overweight and obesity has been increasing worldwide [4]. In Portugal, over 20% of population was considered obese in 2015–2016, and prevalence among the elderly was twice as much [5]. 

These transitions observed in epidemiological profile are due to countries’ rapid urbanization and shifts in the way people commute, eat, exercise, as well as changes in the food system, reflected in food production and processing [6]. In four decades (1974 to 2011) a nutrition transition has been observed in Portugal, revealing a decrease in the consumption of cereals, tubers and pulses supply, along with an increase in meat, dairy, sugar and sweeteners and total fat from animal products, resulting in an increase in energy availability of 406 kcal/person/day [7]. According to the guidelines proposed by the European Food Safety Authority (EFSA) for the European population, one third of adults and almost half of the elderly had low protein intake, whereas excessive saturated fatty acids intake in adults and the elderly reached 54% and 31%, respectively, and 76% of the Portuguese population exceed the maximum tolerated value for sodium intake in 2015–2016 [8].

Changes in the dietary patterns (DPs) of populations worldwide include increasing consumption of ‘convenience’ food and beverages for time-pressured consumers [9], and many studies have been conducted to investigate the impact of these products on diet quality and health, for example with soft drinks and other sweetened beverages [10]. Different approaches on how to identify this food products based on industrial processing have been proposed [11] and one of them, named NOVA, has been the most used in researches of dietary intake assessment nowadays. The NOVA classification system categorizes foods according to the nature, extent, and purpose of processing into four groups: unprocessed or minimally processed foods, processed culinary ingredients, processed foods and ultra-processed foods (UPF), which are defined as ‘formulations of ingredients, mostly for industrial use only, derived from a series of industrial processes’ [12].

There is a growing body of scientific evidence supporting the association of UPF consumption with several adverse health outcomes, such as overweight/obesity, abdominal obesity, dyslipidemia, metabolic syndrome, depression, cardiovascular and cerebrovascular disease, as well as mortality [13]. The impact of ultra-processed foods on diet quality has been observed in national representative samples around the world [14,15,16,17,18] and is one of the reasons for these associations. In the Portuguese population, a higher consumption of UPF in adults has been recently associated with higher total energy intake, dietary energy density, content of carbohydrates and lower dietary density of fiber, sodium and potassium. In both adults and the elderly, a higher consumption of UPF was associated with higher contents of free sugars, total fats and saturated fats, and lower content of protein [19].

The reference to the term “food processing” is occasionally made in the epidemiological literature on modern DPs. Analyses of DP can be performed either by score-based approaches (a priori), such as healthy eating indexes, or using data-driven techniques (a posteriori) [20] and have been used to estimate associations of DP with social, economic, behavioral and health factors. In Portugal, some studies have been conducted, aiming to describe DPs of Portuguese adults and the elderly in the general population, either to relate with social and behavioral factors or with nutritional status, nutrient content of diets and health outcomes [21,22,23,24,25]. However, there is still lack of evidence about DP analyses that consider the nature, extent and purpose of food processing.

The identification and characterization of DPs in populations is useful to identify axes of action for public policies, improve the development of interventions adapted to the needs of specific population groups and decrease the risk of non-communicable diseases. Therefore, this study aims to identify DPs using food groups based on NOVA classification system and their associations with socioeconomic, demographic, lifestyle and nutritional factors in Portuguese adults and the elderly.

## 2. Materials and Methods

### 2.1. Study Design and Subjects

The UPPER project uses data from the National Food, Nutrition and Physical Activity Survey of the Portuguese Population (IAN-AF 2015–2016), on which a protocol and methodology have already been published [26,27]. The IAN-AF was designed to be representative of the Portuguese general population aged between three months and 84 years of age, and its sample was selected by multistage sampling, using the National Heath Registry as a frame, stratified by the seven Statistical Geographic Units: North, Center, Lisbon Metropolitan Area, Alentejo, Algarve and the autonomous regions of Azores and Madeira. Primary health care units and individuals, according to sex and age groups, were randomly selected on each stage of sampling.

For the present study, all adults (18–64 years) and the elderly (65–84 years) who participated in two dietary interviews were included. Thus, total sample was made up of 3852 individuals, including 3102 adults and 750 elderly individuals.

### 2.2. Sociodemographic, Anthropometric, and Physical Activity Data

The IAN-AF data collection was performed by Computer-Assisted Personal Interviewing (CAPI) face-to-face interview, using the “YoueAT&Move” platform, especially developed for the survey. This platform was composed by three modules: “You”, “eAT24” and “Move”, comprising the evaluation of several dimensions [26,27], of which the present study focus on dietary intake and sociodemographic data. In order to lessen seasonal variability, field work lasted for 12 months, and two face-to-face interviews were performed at the participant’s home or at the primary health care unit they belong to, with an interval of 8 to 15 days between each other. 

In the first interview, information about sex (male/female) and birth date, imported from the National Health Registry, were confirmed with the participant. Additionally, other sociodemographic data were obtained at this appointment. Educational level is presented into three categories: none, 1st and 2nd cycle of primary education; 3rd cycle of primary education and high school; and higher education level. Household monthly income was considered as total budget of all household members and classified in ranges for this paper, namely EUR ≤ 970, EUR 971 to 1940 and EUR ≥ 1941. Finally, occupational status was defined as “worker for a fee or profit”, “unemployed” or “other situation” (retired, permanently disabled, student, domestic worker, etc.).

Weight and height were evaluated according to standard procedures [28], by trained personnel. Body weight was measured to the nearest tenth of a kilogram using a digital scale (SECA 813, Hamburg, Germany) and height was measured to the nearest centimeter using a portable wall stadiometer (SECA 213, Hamburg, Germany). BMI (kg/m^2^) was calculated, for which it was assumed the standard cut offs [29], but for descriptive analysis purposes, BMI was classified in three categories: <25.0 kg/m^2^, 25.0 to 29.9 kg/m^2^ and ≥30.0 kg/m^2^. 

Physical activity status was obtained from the “Move” module of platform “You eAT&Move”, through the short version of the International Physical Activity Questionnaire (IPAQ), an instrument for the assessment of physical activity in large population-based studies [30], with seven questions that allow estimating the number of days and time spent, per week, in vigorous and moderate physical activities, walking and sitting time. Subjects were classified as low active, moderately active or highly active, according to the IPAQ scoring protocol [31].

### 2.3. Dietary Assessment and Food Processing Classification

Two non-consecutive 24 h recalls were collected in both the first and second interviews, using the eAT24 module. This software follows the Automated Multiple-Pass Method [32] for 24 h (five steps) to obtain details about each consumed food or beverage including name, quantity, brand and cooking method, as well as the place and time for each eating occasion. When the weight or volume of consumed food item was unknown, food portion size was estimated with the help of an illustration book, household measures list and package information [32]. The eAT24 uses the Portuguese Food Composition Table [33] to estimate energy and nutrients intake from the report of food consumption obtained on the 24 h recalls. 

All reported food and beverages that resulted from a homemade recipe were disaggregated to the level of the ingredients, thus these could be identified given the extent and purpose of food processing according to the NOVA system, of which detailed descriptions can be found elsewhere [12]. Food item classification was independently conducted by two experts in food consumption assessment and in the NOVA system. Afterward, another expert researcher checked the classifications, pinpointing discrepant items to be discussed among all researchers, who performed the classification by consensus. In case of dubious classification, experts deliberated for the most conservative one. 

NOVA classifies foods and beverages into four groups, namely: (1) Unprocessed or minimally processed foods, which are those consumed as obtained in nature or that underwent industrial processes that do not add any substances to the original food, such as drying, boiling, freezing or others, with the objective of extend their shelf life or make their preparation easier, for example, fruits, eggs, fresh meat and frozen vegetables; (2) Processed culinary ingredients, obtained directly from nature or group 1 foods, which are used in the preparation, seasoning and cooking of foods, such as oils and fats, sugar and salt; (3) Processed foods, which are industrial products composed by adding a substance found in group 2 to group 1 foods, usually to increase their durability, and that can include cooking methods for example cheese, breads and salt-cured meat or fish; (4) Ultra-processed foods, defined as formulations of ingredients, mostly of exclusive industrial use, that result from several industrial processes and frequently are added by colors, flavors, emulsifiers and other cosmetic or sensory intensifying additives to make the final product palatable or more appealing. Some examples are soft drinks, confectionery, processed cheese, industrial breads, sausage and other reconstituted meat products [12]. 

The average dietary content of total energy, macro and micronutrients for the whole sample were also calculated, in order to identify differences among DPs. Total energy intake was expressed as kcal/day, dietary energy density was obtained by dividing total energy by total amount consumed, in grams. Dietary content of proteins, carbohydrates, free sugars, total fats and saturated fats were expressed as percentage of total energy intake, whereas dietary content of fiber, vitamins and minerals were expressed as nutrient density (grams, milligrams or micrograms per 1000 kcal). The content of free sugars was estimated using a specific algorithm [34] and previously applied in the IAN-AF 2015–2016, as described elsewhere [35]. 

### 2.4. Dietary Pattern Analysis

All reported food items classified according to the NOVA system were divided into 44 food subgroups, of which contribution in grams (% of grams related to total grams consumed in 24 h) was considered to obtain dietary patterns. To minimize the impact of zero inflation and noncontinuous variables from the 24 h recalls, each food subgroup was divided into categories of consumption, according to the percentage of zeros: food groups that presented less than 20% of zeros were categorized in terciles; food groups that had more than 20% but less than 80% of zeros were also divided in three categories—no consumption, below consumers’ median, above consumers’ median; lastly, food groups that presented more than 80% of zeros were separated in a dichotomous variable, whether subjects consumed or did not consume that food group. 

DPs were derived a posteriori by a latent class analysis model, including sex and age as concomitant variables, using the polytomous outcome variables (poLCA) package for the R language and software environment for statistical computation (version 4.0.3, R Foundation for Statistic Computing, Vienna, Austria, 2020). Models with two to nine latent classes were identified. The number of selected classes (patterns) was decided based on the lower value of the Bayesian Information Criteria (BIC) and substantive interpretation. Subjects were assigned to each pattern according to the highest probability of class membership, and selected DPs were then characterized using weighted prevalence of individuals on extreme categories of consumption of food subgroups for each DP. 

### 2.5. Statistical Analisys

Logistic regression analysis was performed to associate the highest category of consumption of subgroups with each DP membership. Additionally, multinomial logistic regression bivariate and multivariate models were used to obtain crude and adjusted odds ratios (OR) and respective 95% confidence intervals (CI), which were used for the association of DPs with sex, age, typology of neighborhood and educational level. In addition, bivariate and multivariate linear regression models were performed to test for differences on energy and nutrient intake across DPs, with a Sidak adjustment for multiple comparisons. In order to consider the study design effect, all statistical analyses were performed using complex samples analyses on the SPSS statistical software package version 27 (SPSS Inc., Chicago, IL, USA). A significance level of 5% was adopted in all analyses.

## 3. Results

### 3.1. Dietary Patterns

The latent class model with sex and age as concomitant variables extracted three DPs (classes) (two classes, BIC = 293,352.7; three classes, BIC = 291,714.6; four classes, BIC = 291,853.9). Table 1 presents the proportion of subjects within extreme categories of consumption of food subgroups based on NOVA classification: unprocessed or minimally processed foods (1), processed culinary ingredients (2), processed foods (3) and ultra-processed foods (4). The first DP (DP1) had a higher percentage of individuals with the highest consumption of: (1) potatoes and other tubers and roots, vegetables and fungi, eggs, fish and seafood; (2) olive oil, cooking salt and “other processed culinary ingredients”; (3) canned meat or fish, beer and wine, and breads, rice/corn crackers and popcorn. This pattern was thus labelled “Traditional”. On the other hand, DP2 presented the highest frequency of people with a higher consumption of: (1) pasta; (2) plant oils; (3) canned vegetables and legumes (beans), cake and desserts, condensed milk and sweetened yogurt, nectars; (4) carbonated beverages, other sugar-sweetened beverages, distilled alcoholic beverages and flavored ciders, industrial breads and toasts, confectionery, cookies and biscuits, sausages and reconstituted meat products, ready-to-eat and ready-to-heat foods, sauces, dressings and gravies. This pattern was labelled as “Unhealthy”. DP3 stands out for the higher frequency of subjects consuming nuts and seeds, but mostly for presenting the lowest proportion of people in the highest consumption category for (1) cereals, pasta, poultry, red meat; (2) table sugar, plant oil, cooking salt; (3) beer and wine; (4) carbonated and other sugar-sweetened beverages, distilled alcoholic beverages or flavored ciders, sausage and reconstituted meat products. For this reason, DP3 was labelled as “Diet concerns”.

**Figure 1:**
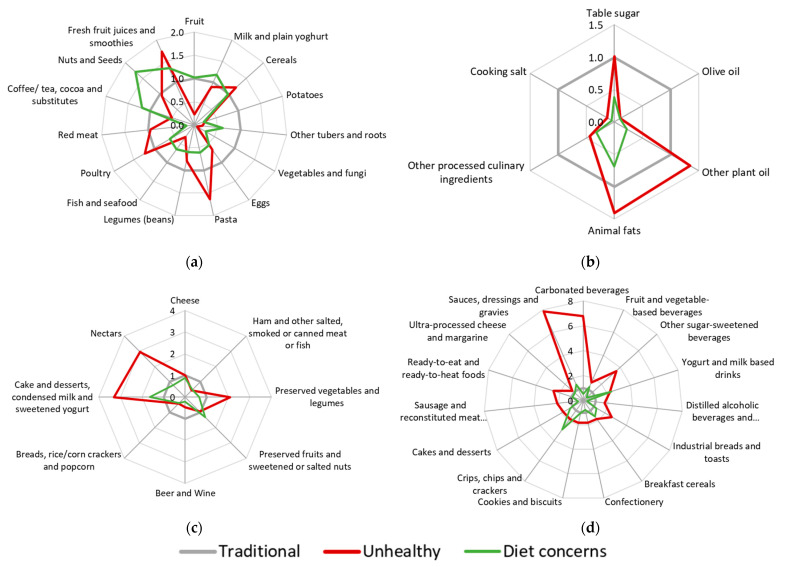
Odds ratio of being in the highest category of consumption of NOVA food subgroups for “Traditional”, “Unhealthy” and “Diet concerns” DPs: (**a**) Unprocessed or minimally processed food; (**b**) Processed culinary ingredients; (**c**) Processed food; (**d**) Ultra-processed food. Traditional DP was used as reference.

In order to provide a visual representation of how the three obtained DPs differ regarding the intake of their components, radar graphs were created using the odds ratio of the category of greater consumption of all subgroups included in latent class analysis (Figure 1). Confidence intervals for the odds ratio are presented in Supplementary Table S1.

### 3.2. Socioeconomic Characteristics, Physical Activity and Nutritional Factors According to Dietary Patterns

The socioeconomic characteristics and nutritional status of subjects according to their DPs are shown in Table 2. Weighted prevalence of people living in the North region was higher on the “Traditional” DP (*p* < 0.001), which also had a lower prevalence of individuals that completed higher education (*p* < 0.001). The “Unhealthy” DP was followed only by adults and presented the lowest age mean (*p* < 0.001), as well as a higher prevalence of those who completed post-secondary education (*p* < 0.001) and those with a BMI < 25.0 kg/m^2^ (*p* < 0.001). The prevalence of females was significantly higher among those on the “Diet concerns” DP (*p* < 0.001), which was also followed by less unemployed subjects (*p* < 0.001).

DPs’ relations with sociodemographic factors are described in Table 3. Adjusted multinomial logistic analysis showed that “Unhealthy” DP was associated with being younger and having more year of education, whereas “Diet concerns” DP was positively associated with being female and negatively associated with having less years of education.

The nutritional intake of subjects following each DP is presented in Table 4. Data are presented as crude and adjusted means for the “Traditional” DP, which was used as reference, besides crude and adjusted coefficients for “Unhealthy” and “Diet concerns” DPs. Therefore, crude and adjusted means for both “Unhealthy” and “Diet concerns” DPs can be obtained by adding its respective coefficients to the “Traditional” DP means; for example, the adjusted mean for the contribution of UPF on the “Unhealthy” DP was 27.10%, which corresponds to the “Traditional” DP mean plus the adjusted coefficient of +10.20. After adjusting for sociodemographic variables, individuals in the “Traditional” pattern reported a significantly higher contribution of processed culinary ingredients, alcohol and sodium intake, and a lower contribution of ultra-processed foods and carbohydrates, compared with those in the other patterns. Using “Traditional” as a reference, “Unhealthy” DP reported a significantly higher intake of energy, saturated fats, free sugars and calcium, and a lower intake of proteins, dietary fiber, vitamin A, vitamin C, folates, potassium, magnesium and iron. Finally, people on the “Diet concerns” DP showed lower total energy and fats, whereas they showed a higher intake of dietary fiber, vitamin C, potassium, calcium, phosphorus, magnesium and zinc compared with “Traditional” DP.

## 4. Discussion

This study was conducted aiming to identify a posteriori-derived DPs in a national representative sample of the Portuguese population over 18 years old and to examine the association of DPs with sociodemographic factors, physical activity, nutritional status and nutrient content of diet. Three DPs emerged from a latent class analysis with sex and age as concomitant variables: “Traditional”, “Unhealthy” and “Diet concerns”, all of them followed by over a third of population. In other studies of person-centered DP approaches, a “processed” or “Western” DP has been followed by 20% [36] to over a third of subjects [25,37], whereas “prudent” or “healthier” DPs tend to be followed by 20% of the population [25,36,37]. In general, the investigation of DPs in other adults and the elderly result in patterns usually named “traditional”, which reflects the most common DP of the population, “unhealthy”, which includes a high consumption of meats and an energy-dense–nutrient-poor diet, and “healthy”, which has a high intake of fruits and vegetables [38]. In the present study, the DP that could correspond with this last point was named “Diet concerns”, once its followers demonstrated some common dietary restrictions of people dieting (less pasta, tubers and beans), with a greater consumption of UPF popularly regarded as healthy, such as crackers and ultra-processed yoghurts, which can reflect the pervasive presence of nutrition and health claims in many UPF packages [39].

Younger people were more likely to follow the “Unhealthy” DP, which was consistently observed in other studies that investigated DPs in the same age groups [21,25,36,37,40,41]. Additionally, in this study, females were more likely to follow the “Diet concerns” DP, which may correspond to a “prudent” DP, that was also strongly associated with being female in other studies [24,36], possibly reflecting differences in dietary habits between genders, such as a higher intake of dairy products, fruits and vegetables among women in comparison with men [42]. Likewise, individuals with a higher level of education were more likely to follow the “Diet concerns” DP when compared with the “Traditional” DP, which was also observed in Portugal [25] and other countries [36,41,43].

With respect to energy and nutrient intake, in the present study, “Unhealthy” DP followers had a higher total energy intake and contributions of UPF, carbohydrates, saturated fats and free sugars, and a lower contribution of unprocessed or minimally processed foods, processed culinary ingredients, and protein, as well as a lower intake of alcohol, dietary fiber, vitamin A, vitamin C, folates, sodium, potassium, magnesium and iron, and a slightly lower energy density than those following the “Traditional” DP. Additionally, compared with followers of “Traditional” DP, subjects following the “Diet concerns” DP presented lower total energy intake, energy density, contribution of processed culinary ingredients and total fats, as well as alcohol, dietary fiber and sodium per 1000 kcal, but higher contribution of UPF, proteins and carbohydrates, vitamin A, vitamin C, potassium, calcium, phosphorus, magnesium and zinc. Although both “Unhealthy” and “Diet concerns” DPs presented a higher contribution of UPF compared with the “Traditional” DP, differences in its profile can be highlighted. Whereas the “Unhealthy” DP is positively associated with a high consumption of sugar-sweetened beverages and meat products, the “Diet concerns” DP was inversely associated with these subgroups, and positively associated with high consumption of milk-based drinks, chips and crackers. 

In general, findings from this study are compatible with those observed in other Portuguese studies, where a better nutrients profile is associated with healthier DPs and where a worse nutrients profile is associated with unhealthier DPs [21,24,25], except for the fact that “Unhealthy” DP showed a lower intake of sodium than “Traditional” DP, whereas unhealthier DPs of other studies usually present a higher intake [24,25]. One possible explanation for this difference is that the “Unhealthy” DP in this study is mainly composed of ultra-processed foods, and in the Portuguese diet, sodium intake mainly came from the added salt of culinary preparation, comprising more than half of the total sodium intake (unprocessed or minimally processed foods plus processed culinary ingredients), followed by processed foods [19]. 

This study had some limitations that should be mentioned. Using a posteriori identification of DPs implies that some subjective decisions were taken by researchers, such as the number of food items (or groups) to include in the analysis and DP labelling. In addition, the sample presented a wide age range, which could affect the derived DPs, since eating habits may be related to age. Additionally, the cross-sectional design makes it impossible to establish causal inferences, since DPs and their possible outcomes were observed at the same time. Finally, dietary intake was estimated by 24 h recalls, a self-reported tool that relies on the respondent’s memory, and the misreporting of food consumption, especially of unhealthy foods, was common.

Regardless of the aforementioned limitations, the present study is original in considering the classification of food items according to the extent and purpose of processing to extract DPs and evaluate its association with sociodemographic factors and the nutrient profile of the diet within a national sample of adults and the elderly. This study adds up to the evidence about DPs of the Portuguese population and contributes findings concerning UPF in this country. Latent class analysis is among one of the most recent methods that have emerged in recent decades for the identification of DPs [44], and is also used to identify other subjects of eating patterns, such as meal consumption patterns [45]. Being a data-driven approach, it may reflect what people eat in daily life and provide interesting insights into dietary behavior [46]. Using age and sex as concomitant variables in latent class analysis made the probabilities of latent class membership conditional on age and sex, which has improved the model’s fitness. Despite the differences in methods for obtaining DPs between this study and others, we have attempted to identify similarities in the literature in order to compare results. Additionally, 24 h recalls were performed by trained researchers, and the multiple-pass method was applied to minimize the omission of possible forgotten foods [32].

## 5. Conclusions

Three DPs were identified in the Portuguese adult population, each of them followed by approximately one-third of the sample. “Traditional” DP had the highest contribution of processed culinary ingredients, alcohol and sodium intake, and the lowest contribution of carbohydrates. In comparison with “Traditional” DP, “Unhealthy” and “Diet concerns” DPs presented a higher contribution of UPF. The “Diet concerns” DP presented a low intake of food items such as pasta, red meat, sugars, alcoholic and sugar-sweetened beverages, but with an expressive contribution of ultra-processed foods, and was followed mostly by women. The “Unhealthy” DP was characterized mostly by the highest consumption of ultra-processed foods, was followed by younger adults, had the worst nutrient profile, and was higher in free sugars and lower in most vitamins and minerals. These findings should be considered when designing and disseminating food-based interventions or guidelines for the prevention of non-communicable diseases in the Portuguese population. 

## Figures and Tables

**Table 1:** Weighted prevalence * of subjects within consumption categories in each dietary pattern—Portuguese population aged 18–84y: The UPPER project.

		DP 1 Traditional	DP 2 Unhealthy	DP 3 Diet Concerns
Food Group	Consumption Category	*n* = 1379 35.8%	*n* = 1256 32.6%	*n* = 1217 31.6%
**Unprocessed or Minimally Processed Foods**				
Fresh fruits	1st tercile	31.6 ^a^	58.0 ^b^	26.2 ^a^
	3rd tercile	40.1 ^a^	13.3 ^b^	40.6 ^a^
Milk and plain yoghurt	No consumption	34.8 ^a^	26.7 ^b^	26.9 ^b^
	≥Median	34.5 ^a^	32.0 ^a^	38.4 ^a^
Cereals	No consumption	26.5 ^a^	13.9 ^b^	36.5 ^c^
	≥Median	45.0 ^a^	49.7 ^a^	22.9 ^b^
Potatoes	No consumption	23.4 ^a^	47.1 ^b^	52.6 ^b^
	≥Median	50.1 ^a^	19.9 ^b^	17.6 ^b^
Other tubers and roots	No consumption	14.6 ^a^	28.5 ^b^	18.1 ^a^
	≥Median	55.1 ^a^	18.1 ^b^	43.2 ^c^
Vegetables and fungi	No consumption	11.3 ^a^	57.8 ^b^	33.1 ^c^
	≥Median	58.8 ^a^	9.5 ^b^	28.9 ^c^
Eggs	No consumption	44.2 ^a^	32.9 ^b^	58.6 ^c^
	≥Median	35.0 ^a^	26.0 ^b^	22.4 ^b^
Pasta	No consumption	63.0 ^a^	46.2 ^b^	69.3 ^a^
	≥Median	19.8 ^a^	28.7 ^b^	13.1 ^c^
Legumes (beans)	No consumption	54.3 ^a^	51.5 ^a^	65.1 ^b^
	≥Median	24.1 ^a^	20.1 ^a,b^	15.9 ^b^
Fish and seafood	No consumption	48.5 ^a^	60.2 ^a^	52.7 ^a^
	≥Median	33.1 ^a^	13.5 ^b^	24.2 ^c^
Poultry	No consumption	44.3 ^a^	34.1 ^b^	47.5 ^a^
	≥Median	29.7 ^a^	34.1 ^a^	20.2 ^b^
Red meat	No consumption	21.1 ^a^	17.5 ^a^	46.9 ^b^
	≥Median	48.1 ^a^	46.8 ^a^	13.9 ^b^
Coffee/tea, cocoa and substitutes	No consumption	35.3 ^a^	43.0 ^a^	36.9 ^a^
	≥Median	32.7 ^a,b^	19.7 ^a^	36.5 ^b^
Nuts and Seeds	No consumption	85.7 ^a^	86.4 ^a^	77.8 ^b^
	Consumption	14.3 ^a^	13.6 ^a^	22.2 ^b^
Fresh fruit juices and smoothies	No consumption	78.2 ^a^	73.0 ^a^	78.8 ^a^
	<Median	13.0 ^a^	12.6 ^a^	9.7 ^a^
	≥Median	8.8 ^a^	14.3 ^a^	11.5 ^a^
**Processed Culinary Ingredients**				
Table sugar (Honey, molasses, syrups)	No consumption	21.4 ^a^	20.4 ^a^	41.5 ^b^
	≥Median	44.2 ^a^	44.4 ^a^	23.2 ^b^
Olive oil	1st tercile	6.0 ^a^	35.9 ^b^	54.1 ^c^
	3rd tercile	68.7 ^a^	19.2 ^b^	17.8 ^b^
Other plant oil	No consumption	45.2 ^a^	22.5 ^b^	64.0 ^c^
	≥Median	34.5 ^a^	41.6 ^b^	10.3 ^c^
Animal fats	No consumption	51.2 ^a^	36.6 ^b^	61.3 ^c^
	≥Median	24.6 ^a^	31.4 ^a^	18.4 ^a^
Other processed culinary ingredients (vinegar, gelatin)	No consumption	33.8 ^a^	36.6 ^a^	53.0 ^b^
	≥Median	42.8 ^a^	24.7 ^b^	19.5 ^b^
Cooking salt	1st tercile	3.4 ^a^	32.5 ^b^	58.1 ^c^
	3rd tercile	69.7 ^a^	22.6 ^b^	9.7 ^c^
**Processed Foods**				
Cheese	No consumption	49.5 ^a^	28.8 ^b^	41.4 ^a^
	≥Median	29.0 ^a^	29.2 ^a^	26.6 ^a^
Ham and other salted, smoked or canned meat or fish	No consumption	55.4 ^a^	65.1 ^b^	64.9 ^b^
	≥Median	28.7 ^a^	14.5 ^b^	15.8 ^b^
Preserved vegetables and legumes	No consumption	43.6 ^a^	24.9 ^b^	56.9 ^c^
	≥Median	25.6 ^a^	41.6 ^b^	18.1 ^a^
Preserved fruits and sweetened or salted nuts	No consumption	86.7 ^a^	87.2 ^a^	83.3 ^a^
	Consumption	13.3 ^a^	12.8 ^a^	16.7 ^a^
Beer and wine	No consumption	15.2 ^a^	13.3 ^a^	43.3 ^b^
	≥Median	54.2 ^a^	35.7 ^b^	19.3 ^c^
Breads, rice/corn crackers and popcorn	1st tercile	28.5 ^a^	46.0 ^b^	44.9 ^b^
	3rd tercile	40.4 ^a^	23.1 ^b^	20.9 ^b^
Cake and desserts, condensed milk and sweetened yogurt	No consumption	95.6 ^a^	86.9 ^b^	93.0 ^a^
	Consumption	4.4 ^a^	13.1 ^b^	7.0 ^a^
Nectars	No consumption	93.4 ^a^	82.7 ^b^	94.7 ^a^
	Consumption	6.6 ^a^	17.3 ^b^	5.3 ^a^
**Ultra-Processed Foods**				
Carbonated beverages	No consumption	91.1 ^a^	59.9 ^b^	94.8 ^c^
	Consumption	8.9 ^a^	40.1 ^b^	5.2 ^c^
Fruit and vegetable-based beverages	No consumption	89.2 ^a^	83.6 ^a^	87.2 ^a^
	Consumption	10.8 ^a^	16.4 ^a^	12.8 ^a^
Other sugar-sweetened beverages	No consumption	88.4 ^a^	68.2 ^b^	95.4 ^c^
	≥Median	11.6 ^a^	31.8 ^b^	4.6 ^c^
Yogurt and milk-based drinks	No consumption	63.0 ^a^	32.5 ^b^	36.6 ^b^
	≥Median	18.3 ^a^	33.4 ^b^	34.8 ^b^
Distilled alcoholic beverages and flavored ciders	No consumption	86.8 ^a^	79.2 ^b^	95.4 ^c^
	Consumption	13.2 ^a^	20.8 ^b^	4.6 ^c^
Industrial breads and toasts	No consumption	78.7 ^a^	49.8 ^b^	67.3 ^c^
	≥Median	11.8 ^a^	26.0 ^b^	14.1 ^a^
Breakfast cereals	No consumption	89.5 ^a^	74.4 ^b^	75.9 ^b^
	≥Median	6.9 ^a^	12.0 ^a^	10.5 ^a^
Confectionery	No consumption	79.4 ^a^	58.4 ^b^	79.5 ^a^
	≥Median	11.5 ^a^	19.1 ^b^	8.8 ^a^
Cookies and biscuits/Packaged sweet snacks	No consumption	67.4 ^a^	50.8 ^b^	59.1 ^a,b^
	≥Median	16.3 ^a^	26.1 ^b^	16.0 ^a^
Crips, chips and crackers/Packaged savory snacks	No consumption	92.8 ^a^	87.6 ^a,b^	81.8 ^b^
	Consumption	7.2 ^a^	12.4 ^a,b^	18.2 ^b^
Cakes and desserts	No consumption	72.2 ^a^	48.2 ^b^	66.7 ^a^
	≥Median	15.2 ^a^	24.8 ^b^	18.1 ^a,b^
Sausage and reconstituted meat products	No consumption	32.4 ^a^	13.2 ^b^	44.7 ^c^
	≥Median	35.3 ^a^	53.3 ^b^	18.0 ^c^
Ready-to-eat and ready-to-heat foods	No consumption	84.3 ^a^	68.0 ^b^	85.7 ^a^
	Consumption	15.7 ^a^	32.0 ^b^	14.3 ^a^
Ultra-processed cheese, margarine and other spreads	No consumption	62.4 ^a^	56.8 ^a^	61.3 ^a^
	≥Median	18.3 ^a^	20.8 ^a^	17.1 ^a^
Sauces, dressings and gravies	No consumption	80.1 ^a^	53.1 ^b^	83.6 ^a^
	≥Median	4.9 ^a^	28.9 ^b^	6.9 ^a^

Two classes, BIC = 293,352.7; three classes, BIC = 291,714.6; four classes, BIC = 291,853.9; * Intermediate categories (2nd tercile or below median) were not shown in order to avoid redundancy; different superscript letters indicate significant differences between dietary patterns within categories of consumption at a significance level of 5%.

**Table 2:** Sociodemographic characteristics, physical activity and BMI according to dietary patterns among Portuguese population aged 18–84: The UPPER project.

	*n*	DP 1 Traditional *n* = 1379 35.8%	DP 2 Unhealthy *n* = 1256 32.6%	DP 3 Diet Concerns *n* = 1217 31.6%
		% (95% CI)	% (95% CI)	% (95% CI)
**Sex**				
Female	2032	41.7 (39.0–44.4)	42.2 (38.7–45.8)	**76.8 (73.0–80.2)**
Male	1820	58.3 (55.6–61.0)	57.8 (54.2–61.3)	**23.2 (19.8–27.0)**
**Age group**				
Adults (18–64 years)	3102	71.8 (68.7–74.7)	100	66.5 (62.3–70.4)
Elderly (65–84 years)	750	28.2 (25.3–31.3)	-	33.5 (29.6–37.7)
Age (years)–mean (CI 95%)	-	54.5 (53.4–55.6)	**36.62 (35.6–37.6)**	53.1 (51.6–54.6)
**Region**				
North	651	**42.1 (38.1–46.2)**	32.8 (28.6–37.2)	29.1 (23.5–35.5)
Centre	669	23.5 (21.1–26.0)	20.4 (16.9–24.5)	22.3 (18.3–26.7)
Lisbon MA	525	21.5 (18.4–25.0)	30.2 (26.1–34.6)	29.0 (24.5 (33.9)
Alentejo	463	5.8 (4.4–7.5)	6.3 (5.0–7.9)	8.4 (6.6–10.6)
Azores	508	1.6 (1.2–2.1)	3.3 (2.0–5.6)	2.5 (1.8–3.6)
Madeira	515	2.3 (2.0–2.6)	2.4 (1.7–3.4)	3.4 (2.5–4.5)
Algarve	521	3.2 (2.4–4.3)	4.6 (3.6–5.8)	5.3 (4.1–6.9)
**Typology of the neighborhood**				
Predominantly rural area	369	10.0 (5.9–16.6)	7.7 (4.1–14.1)	7.3 (4.1–12.7)
Medium urban area	680	15.2 (8.8–25.1)	13.5 (8.1–21.6)	12.0 (7.0–19.6)
Predominantly urban area	2803	74.8 (65.4–82.3)	78.8 (70.7–85.1)	80–7 (73.6–86.3)
**Education**				
None/primary education	1342	45.2 (40.3–50.2)	**11.8 (9.4–14.8)**	37.1 (32.6–41.8)
Secondary/post-secondary education	1629	38.6 (34.4–42.9)	**60.9 (55.5–66.0)**	37.2 (33.6–40.9)
Higher education	876	**16.2 (12.8–20.3)**	27.3 (22.7–32.4)	25.7 (21.5–30.5)
**Monthly household income**				
EUR ≤970	1377	43.6 (38.8–48.5)	29.1 (25.6–32.9)	38.8 (34.3–43.6)
EUR 971–1940	1389	37.0 (33.0–41.1)	42.8 (37.8–47.9)	40.2 (35.7–45.0)
EUR ≥1941	708	19.5 (15.5–24.1)	28.1 (23.8–32.9)	20.9 (16.9–25.6)
**Occupational status**				
Employed	2119	47.3 (43.8–50.7)	**68.1 (63.9–72.1)**	48.0 (44.3–51.7)
Unemployed	444	13.2 (10.6–16.3)	13.6 (11.2–16.5)	**7.4 (5.6–9.8)**
Retired, permanently disabled or other	1286	39.6 (35.6–43.7)	18.3 (15.4–21.5)	44.6 (40.4–48.8)
**Body mass index**				
<25.0 kg/m^2^	1340	30.5 (26.5–34.8)	**50.5 (46.1–54.9)**	33.4 (29.0–38.2)
25.0 to 29.9 kg/m^2^	1373	41.1 (36.7–45.6)	**31.0 (27.2–35.0)**	39.7 (35.6–43.9)
≥30.0 kg/m^2^	950	28.4 (24.7–32.5)	**18.5 (15.5–21.9)**	26.9 (23.7–30.4)
kg/m^2^–mean (CI 95%)	-	27.7 (27.3–28.1)	25.8 (25.4–26.2)	27.6 (27.2–28.1)
**Physical activity level**				
Low	1623	44.2 (39.6–48.8)	44.3 (39.9–48.8)	41.9 (37.0–47.0)
Moderate	1176	30.7 (27.0–34.7)	27.2 (23.2–31.6)	33.5 (29.1–38.3)
High	937	25.1 (20.8–30.0)	28.5 (24.8–32.5)	24.5 (20.2–29.4)

Statistically significant differences (by confidence intervals) are highlighted in bold.

**Table 3:** Multinomial logistic regression analysis of the associations between sociodemographic and dietary patterns among Portuguese population aged 18–84 (Unhealthy and Diet concerns vs. Traditional): The UPPER project.

	Unhealthy *n* = 1256 32.6%		Diet Concerns *n* = 1217 31.6%	
	Crude OR (95% CI)	Adjusted OR ^‡^ (95% CI)	Crude OR (95% CI)	Adjusted OR ^‡^ (95% CI)
**Sex**				
Female	1.02 (0.84–1.23)	0.92 (0.72–1.18)	**4.63 (3.59–5.98)**	**4.58 (3.57–5.87)**
Male	1	1	1	1
**Age (years)**	**0.92 (0.92–0.93)**	**0.93 (0.92–0.94)**	0.99 (0.99–1.00)	1.00 (0.99–1.01)
**Typology of the neighborhood**				
Predominantly rural area	0.73 (0.53–1.01)	0.79 (0.56–1.11)	0.68 (0.45–1.02)	0.77 (0.48–1.24)
Medium urban area	0.84 (0.62–1.15)	0.86 (0.60–1.23)	0.73 (0.45–1.18)	0.84 (0.52–1.37)
Predominantly urban area	1			
**Education**				
None/primary education	**0.16 (0.12–0.21)**	**0.51 (0.35–0.74)**	**0.52 (0.38–0.71)**	**0.52 (0.36–0.75)**
Secondary/post-secondary education	0.94 (0.74–1.18)	0.93 (0.71–1.23)	**0.61 (0.44–0.83)**	**0.64 (0.46–0.88)**
Higher education	1	1	1	1

Statistically significant associations are highlighted in bold; ORs presented have the Traditional dietary pattern (*n* = 1379; 35.8%) as the reference category (the ORs = 1 are not shown for brevity); ‡ Models are adjusted for all other variables on the table.

**Table 4:** Nutritional intake according to dietary patterns (DPs) derived by latent class analysis among Portuguese population aged 18–84: The UPPER project.

	Traditional		Unhealthy		Diet Concerns	
	Crude Mean (SE)	Adjusted Mean ^a^ (SE)	Crude Coefficient (*p*-Value)	Adjusted Coefficient ^a^ (*p*-Value)	Crude Coefficient (*p*-Value)	Adjusted Coefficient ^a^ (*p*-Value)
Total energy intake (kcal)	1864.09 (27.59)	1847.04 (28.05)	+308.25 (<0.001)	+233.99 (<0.001)	−430.543 (<0.001)	−264.13 (<0.001)
Energy density (kcal/grams)	0.81 (0.01)	0.80 (0.01)	−0.03 (0.028)	−0.03 (0.010)	−0.24 (<0.001)	−0.22 (<0.001)
Unprocessed or minimally processed foods (% kcal)	44.08 (0.50)	44.01 (0.63)	−6.36 (<0.001)	−7.22 (<0.001)	−0.30 (0.679)	−0.40 (0.584)
Processed culinary ingredients (% kcal)	13.69 (0.28)	13.45 (0.33)	−2.55 (<0.001)	−2.14 (<0.001)	−3.79 (<0.001)	−4.43 (<0.001)
Processed foods (% kcal)	26.50 (0.52)	25.64 (0.67)	−5.66 (<0.001)	−0.84 (0.291)	−2.64 (<0.001)	−0.09 (0.888)
Ultra-processed foods (% kcal)	15.73 (0.34)	16.90 (0.47)	+14.57 (<0.001)	+10.20 (<0.001)	+6.73 (<0.001)	+4.91 (<0.001)
Proteins (% of total energy intake)	18.34 (0.17)	18.50 (0.17)	−0.01 (0.954)	−0.87 (<0.001)	+0.66 (0.009)	+0.76 (0.003)
Carbohydrates (% of total energy intake)	45.84 (0.49)	46.17 (0.44)	+1.29 (0.021)	+1.58 (0.005)	+3.95 (<0.001)	+3.00 (<0.001)
Fats (% of total energy intake)	30.12 (0.39)	30.28 (0.38)	+1.39 (0.002)	+0.28 (0.527)	−0.78 (0.111)	−1.79 (<0.001)
Saturated fats (% of total energy intake)	8.82 (0.13)	8.91 (0.14)	+2.00 (<0.001)	+1.53 (<0.001)	+0.65 (<0.001)	+0.38 (0.047)
Free sugars (% of total energy intake)	6.14 (0.18)	6.60 (0.19)	+4.83 (<0.001)	+3.83 (<0.001)	+0.96 (0.001)	+0.48 (0.106)
Alcohol (g/1000 kcal)	8.13 (0.48)	7.20 (0.49)	−3.81 (<0.001)	−1.43 (0.014)	−5.48 (<0.001)	−2.80 (<0.001)
Dietary fiber (g/1000 kcal)	10.42 (0.12)	10.48 (0.13)	−2.17 (<0.001)	−1.77 (<0.001)	+1.71 (<0.001)	+1.28 (<0.001)
Vitamin A (mcg/1000 kcal)	496.40 (20.59)	491.94 (19.11)	−130.95 (<0.001)	−106.52 (<0.001)	+47.90 (0.095)	+2.53 (0.935)
Vitamin C (mg/1000 kcal)	57.96 (1.29)	59.49 (1.40)	−14.08 (<0.001)	−15.29 (<0.001)	+13.36 (<0.001)	+9.95 (0.005)
Folates (mcg/1000 kcal)	123.69 (1.24)	126.56 (1.54)	−20.57 (<0.001)	−20.90 (<0.001)	+18.96 (<0.001)	+13.82 (<0.001)
Sodium (mg/1000 kcal)	1890.32 (18.14)	1923.26 (18.90)	−280.61 (<0.001)	−271.22 (<0.001)	−212.71 (<0.001)	−220.91 (<0.001)
Potassium (mg/1000 kcal)	1799.57 (14.07)	1806.48 (13.94)	−299.26 (<0.001)	−268.30 (<0.001)	+145.10 (<0.001)	+121.85 (<0.001)
Calcium (mg/1000 kcal)	368.04 (5.38)	379.70 (5.31)	+15.491 (0.062)	+23.22 (0.009)	+171.30 (<0.001)	+152.92 (<0.001)
Phosphorus (mg/1000 kcal)	652.77 (5.76)	662.56 (5.27)	−6.03 (0.381)	−13.09 (0.063)	+80.11 (<0.001)	+71.07 (<0.001)
Magnesium (mg/1000 kcal)	154.66 (1.46)	155.69 (1.35)	−13.78 (<0.001)	−12.74 (<0.001)	+24.55 (<0.001)	+22.04 (<0.001)
Iron (mg/1000 kcal)	6.52 (0.10)	6.55 (0.11)	−0.89 (<0.001)	−0.77 (<0.001)	−0.12 (0.392)	−0.08 (0.580)
Zinc (mg/1000 kcal)	5.28 (0.07)	5.26 (0.08)	+0.09 (0.303)	−0.13 (0.264)	+0.50 (<0.001)	+0.47 (<0.001)

^a^ Adjusted for age, sex, typology of neighborhood and educational level.

## Data Availability

All data generated or analyzed during this study are available from the corresponding author on reasonable request.

## Supplementary Materials

The following are available online at https://www.mdpi.com/article/10.3390/nu13114119/s1, Table S1—Odds ratio of being in the highest category of consumption of NOVA food subgroups for “Unhealthy” and “Diet concerns” dietary patterns.
