# Supplementary material for: An Ultra-Processed Food Dietary Pattern Is Associated with Lower Diet Quality in Portuguese Adults and the Elderly: The UPPER Project
Source: Nutrients. 2021 Nov 17;13(11):4119. doi: 10.3390/nu13114119 (PMC8619325; doi:10.3390/nu13114119)
Supplement: Supplementary file 1 [file nutrients-13-04119-s001.zip › nutrients-1453408-supplementary.pdf]

Supplementary Materials:

Table S1. Odds ratio of being in the highest category of consumption of NOVA food subgroups for "Unhealthy" and "Diet concerns" dietary patterns (*versus* "Traditional" DP) among Portuguese population aged 18–84: The UPPER project.

	**Unhealthy DP**	**Diet concerns DP**
	Odds ratio (95% CI)	Odds ratio (95% CI)
**Unprocessed or minimally processed foods**		
Fruit	**0.228 (0.179 - 0.290)**	1.021 (0.770 - 1.355)
Milk and plain yoghurt	0.893 (0.674 - 1.183)	1.182 (0.929 - 1.503)
Cereals	1.206 (0.975 - 1.491)	**0.362 (0.285 - 0.460)**
Potatoes	**0.248 (0.192 - 0.321)**	**0.213 (0.162 - 0.281)**
Other tubers and roots	**0.18 (0.139 - 0.234)**	**0.619 (0.499 - 0.768)**
Vegetables and fungi	**0.074 (0.055 - 0.099)**	**0.284 (0.222 - 0.364)**
Eggs	**0.654 (0.493 - 0.867)**	**0.538 (0.386 - 0.750)**
Pasta	**1.635 (1.182 - 2.263)**	**0.612 (0.447 - 0.839)**
Legumes (beans)	0.791 (0.587 - 1.067)	**0.597 (0.448 - 0.797)**
Fish and seafood	**0.316 (0.241 - 0.413)**	**0.645 (0.504 - 0.826)**
Poultry	1.226 (0.967 - 1.553)	**0.602 (0.456 - 0.795)**
Red meat	0.947 (0.757 - 1.184)	**0.174 (0.134 - 0.226)**
Coffee/ tea, cocoa and substitutes	**0.505 (0.383 - 0.667)**	1.184 (0.918 - 1.527)
Nuts and Seeds	0.937 (0.660 - 1.331)	**1.704 (1.255 - 2.314)**
Fresh fruit juices and smoothies	**1.724 (1.129 - 2.633)**	1.342 (0.871 - 2.069)
**Processed culinary ingredients**		
Table sugar [Honey, molasses, syrups]	1.010 (0.798 - 1.278)	**0.381 (0.306 - 0.475)**
Olive oil	**0.109 (0.082 - 0.145)**	**0.099 (0.070 - 0.138)**
Other plant oil	**1.354 (1.047 - 1.751)**	**0.218 (0.156 - 0.305)**
Animal fats	**1.403 (1.109 - 1.775)**	**0.689 (0.517 - 0.919)**
Other processed culinary ingredients [vinegar, gelatin]	**0.439 (0.352 - 0.548)**	**0.324 (0.249 - 0.423)**
Cooking salt	**0.127 (0.096 - 0.168)**	**0.047 (0.033 - 0.065)**
**Processed foods**		
Cheese	1.012 (0.772 - 1.325)	0.889 (0.693 - 1.140)
Ham and other salted, smoked or canned meat or fish	**0.422 (0.304 - 0.585)**	**0.467 (0.351 - 0.620)**
Preserved vegetables and legumes	**2.072 (1.652 - 2.599)**	**0.643 (0.465 - 0.887)**
Preserved fruits and sweetened or salted nuts	0.956 (0.697 - 1.311)	1.310 (0.965 - 1.776)
Beer and Wine	**0.470 (0.360 - 0.615)**	**0.203 (0.157 - 0.262)**
Breads, rice/corn crackers and popcorn	**0.442 (0.343 - 0.571)**	**0.390 (0.297 - 0.511)**
Cake and desserts, condensed milk and sweetened yogurt	**3.310 (2.349 - 4.665)**	**1.640 (1.031 - 2.610)**
Nectars	**2.967 (1.903 - 4.624)**	0.788 (0.491 - 1.265)
**Ultra-processed foods**		
Carbonated beverages	**6.803 (5.146 - 8.994)**	**0.556 (0.381 - 0.812)**
Fruit and vegetable-based beverages	**1.614 (1.126 - 2.314)**	1.203 (0.866 - 1.670)
Other sugar-sweetened beverages	**3.572 (2.691 - 4.742)**	**0.367 (0.230 - 0.585)**
Yogurt and milk-based drinks [Includes soy-, whey- based drinks, lactose free drinks, flavoured milk and shakes]	**2.233 (1.731 - 2.881)**	**2.377 (1.886 - 2.995)**
Distilled alcoholic beverages and flavoured ciders	**1.722 (1.25 - 2.373)**	**0.316 (0.207 - 0.481)**
Industrial breads and toasts	**2.621 (2.003 - 3.428)**	1.226 (0.930 - 1.617)
Breakfast cereals	**1.828 (1.073 - 3.115)**	1.579 (0.971 - 2.569)

Confectionery [Includes all candy, chocolate, toffee, caramel, gelatin-based fruit sweets]	**1.821 (1.357 - 2.444)**	0.747 (0.499 - 1.117)
Cookies and biscuits/ Packaged sweet snacks	**1.814 (1.367 - 2.407)**	0.976 (0.651 - 1.462)
Crips, chips and crackers/ Packaged savory snacks	**1.830 (1.196 - 2.800)**	**2.888 (1.922 - 4.339)**
Cakes and desserts [Includes pastries, rolls, ice-cream, muffins, brownies, puddings, tarts, sweet pies, jelly]	**1.834 (1.307 - 2.571)**	1.234 (0.888 - 1.713)
Sausage and reconstituted meat products [Includes nuggets, spam, quorn, fish or crab sticks, plant-based meat substitutes]	**2.091 (1.683 - 2.598)**	**0.402 (0.300 - 0.539)**
Ready-to-eat and ready-to-heat foods	**2.534 (1.956 - 3.282)**	0.897 (0.664 - 1.212)
Ultra-processed cheese, margarine and other spreads	1.166 (0.878 - 1.547)	0.916 (0.686 - 1.223)
Sauces, dressings and gravies [Includes bouillon cubes, gravy granules, all kinds of salad dressing]	**7.858 (5.586 - 11.056)**	1.428 (0.907 - 2.248)

Statistically significant associations are highlighted in bold; ORs presented have the Traditional dietary pattern (*n* = 1379; 35.8%%) as the reference category (the ORs = 1 are not shown for brevity).
